# Acromegaly and the Colon: Scoping Beyond the Pituitary

**DOI:** 10.7759/cureus.20018

**Published:** 2021-11-29

**Authors:** Gautami S Patel, Idan Grossmann, Kevin Rodriguez, Mridul Soni, Pranay K Joshi, Saawan C Patel, Devarashetty Shreya, Diana I Zamora, Ibrahim Sange

**Affiliations:** 1 Internal Medicine, Pramukhswami Medical College, Karamsad, IND; 2 Research, Medical University of Silesia in Katowice Faculty of Medical Sciences Katowice, Katowice, POL; 3 Research, Universidad Americana (UAM) Facultad de Medicina, Managua, NIC; 4 Research, Shri Lal Bahadur Shastri Government Medical College, Mandi, IND; 5 Research, Department of Medicine, B.J. Medical College, Ahmedabad, IND; 6 Medicine, Pramukhswami Medical College, Karamsad, IND; 7 Internal Medicine, Gandhi Medical College, Secunderabad, IND; 8 General Medicine, Universidad de Ciencias Médicas Andrés Vesalio Guzman, San José, CRI; 9 Research, K. J. Somaiya Medical College, Mumbai, IND

**Keywords:** growth hormone, insulin-like growth factor 1, colorectal carcinoma, colorectal polyps, acromegaly

## Abstract

Acromegaly is a complex endocrinological disorder commonly caused by hypersecretion of growth hormone (GH) typically due to pituitary gland tumors. Patients with acromegaly who are successfully treated and biochemically managed have a reasonably average life expectancy. However, it causes a cascade of multi-systemic involvement throughout the patient’s life, including cardiovascular, neuropsychiatric, respiratory, metabolic, neurological, neoplastic, and gastrointestinal involvement, resulting in a higher rate of hospitalization, lower quality of life, and a shorter life expectancy. Although cardiovascular complications are the primary cause of death in patients with acromegaly, malignancy is now emerging as a major killer in these individuals. Colorectal carcinoma has been reported to be prevalent in acromegaly individuals. This review article has compiled studies to demonstrate a link between acromegaly and colorectal neoplasia, intending to provide a strong foundation for their clinical relationship. This article has summarised a potential pathogenic mechanism and provided insights into the clinical presentation of such patients. Furthermore, this article has provided a brief overview of current screening recommendations for colorectal neoplasia in acromegaly patients.

## Introduction and background

Acromegaly is an endocrinological disorder characterized by an abnormally elevated growth hormone (GH) level in the serum predominantly caused by a pituitary adenoma [1,2]. Pierre Marie, a French neurologist, coined the term “acromegaly” to describe this disease’s morphological features [3]. It is a rare disease with an incidence rate of 0.2 to 1.1 per 100,000 people and a prevalence rate ranging between 2.8 and 13.7 cases per 100,000 people. Most patients are diagnosed in their 50s with an average diagnostic delay of 4.5-5 years [4]. Ninety-five percent of the cases are sporadic, and 50% of the cases are present in childhood as a part of familial diseases such as familial isolated pituitary adenoma (FIPA), X linked acrogigantism (XLAG), multiple endocrinal neoplasia-1 and 4 (MEN-1 and MEN-4), Carney complex, McCune-Albright syndrome, neurofibromatosis or ‘3PAs’ syndrome [5]. When GH enters blood circulation, it signals the liver to produce another hormone, called insulin-like growth factor-1 (IGF-1) which mediates many GH effects [6]. While gigantism occurs due to excess GH levels before epiphyseal closure leading to an abnormal linear overgrowth of bones, acromegaly manifests after the epiphyseal closure presenting with morphological features like broad hands, feet, and fingers, wide and thick nasal bones, prominent zygomatic arch, bulging forehead occasionally leading to frontal bossing, swollen lips with marked facial lines due to soft tissue thickening, dental malocclusion due to mandibular overgrowth with prognathism along with maxillary widening leading to tooth separation [6,7]. In addition, visual problems and headaches are believed to be due to the mass effect of pituitary overgrowth [7]. Besides the musculoskeletal system, GH and IGF-1 have several systemic manifestations, including cardiovascular, neuropsychiatric, respiratory, metabolic, neurological, neoplastic, and gastrointestinal complications [7,8]. 

Although the clinical presentation of acromegaly is relatively apparent with regards to the physical appearance of the patients, the workup usually begins with an elevated IGF-1 level as it is an indicator of GH function. It is confirmed with an unsuppressed GH concentration after an oral glucose tolerance test (OGTT) [9]. Radiological investigation such as magnetic resonance imaging (MRI) is often necessary to look for the pituitary adenoma [9]. In certain rare clinical scenarios, Computed tomography (CT) scan of the thorax and abdomen is done to localize towards an ectopic source of secretion of GH or growth hormone-releasing hormone (GHRH) [10]. Even though transsphenoidal surgery is considered the mainstay of therapy, medical management with drugs like octreotide (somatostatin analog), cabergoline (dopamine agonists), pegvisomant (GH receptor antagonist) has been shown to help keep IGF-1 levels in the normal range [11-13]. Acromegaly is a complex disorder that manifests with multiple system involvement, out of which cardiovascular complications (congestive heart failure) are the main culprit behind the mortality in these patients [14]. One of the lesser-explored areas in the subject of acromegaly is that of colonic involvement, which can manifest with conditions such as colorectal polyps and carcinoma [15]. These conditions exhibit a subtle clinical course. Most of them have an asymptomatic and insidious presentation, resulting in an average of 12 years of delay in diagnosis [1]. This could result in the development of colon polyps and possibly allow for pre-malignant lesions to transform into cancer, further amplifying the patient’s mortality. This review article aims to: 1. Underline the pathogenesis and explore the clinical relationship between acromegaly and the development of colonic complications; and 2. Emphasize the importance of early screening and diagnosis of these conditions.

## Review

Acromegaly and colorectal polyps

**Figure 1:**
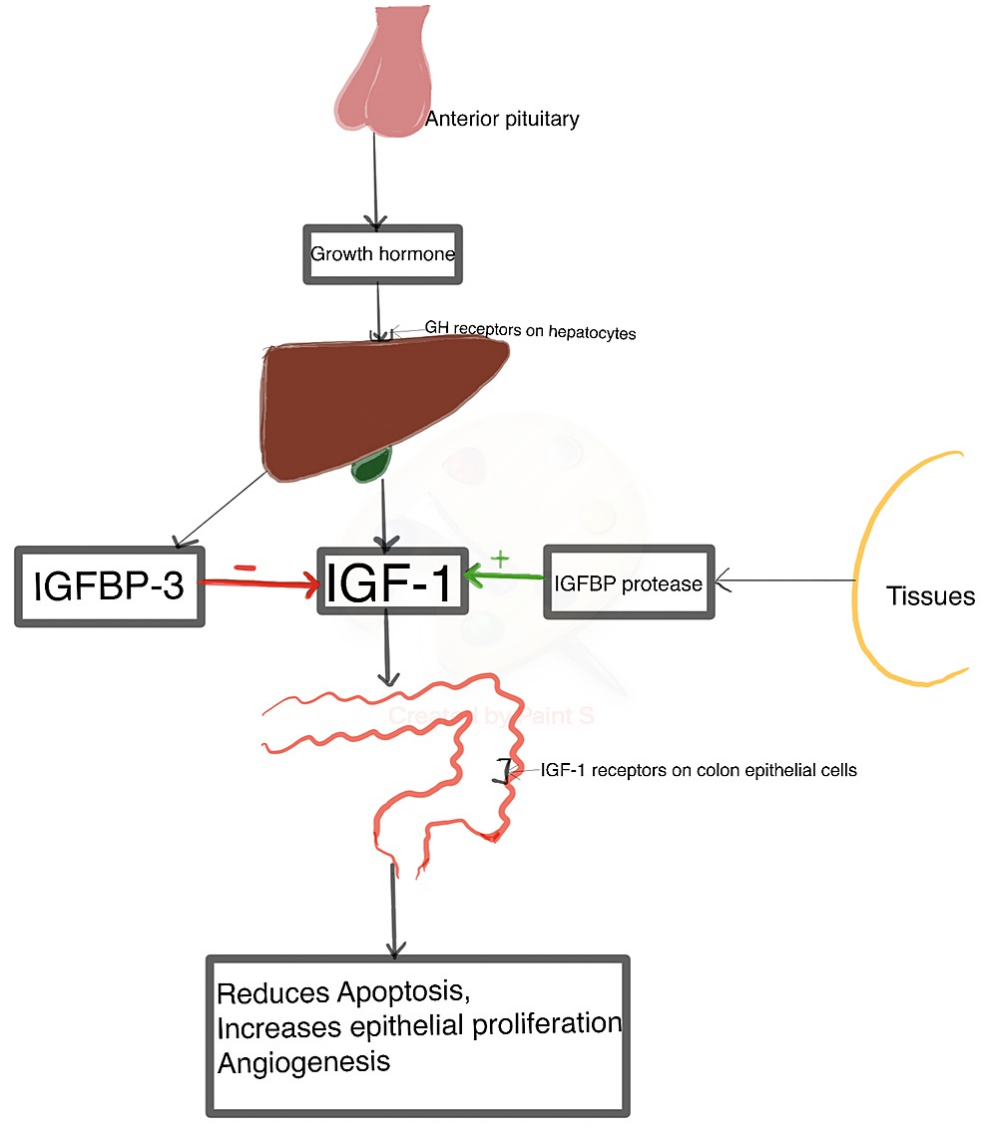
A summary of the possible pathogenesis of acromegaly that results in colorectal complications. GH: Growth Hormone IGFBP-3: Insulin-like Growth Factor Binding Protein-3 IGF-1: Insulin-like Growth Factor-1

Acromegaly is most commonly caused by an anterior pituitary somatotrophic tumor that secretes GH, also known as somatotropin [1]. GH is a protein hormone that binds to its membrane-bound growth hormone receptor (GHR) on the liver [1,2]. Activated GHR, in turn, activates the enzyme Janus kinase 2 (JAK-2), a cytoplasmic tyrosine kinase that phosphorylates tyrosine residues both within the JAK-2 enzyme and GHR [1,2,16]. That serves as binding sites for a variety of signaling molecules leading to alteration of gene expression [16]. As a result, the liver secretes IGF-1, also known as somatomedin, which binds to its receptor on the colonic epithelium and serves its role (Figure 1) [1,2]. The amounts of insulin-like growth factor binding protein-3 (IGFBP-3) and IGFBP protease in the blood affect the circulating levels of IGF-1. IGFBP-3 is produced by the liver alongside IGF-1 and works to inhibit IGF-1 activity by binding to it and lowering its free levels [16]. Tissue produces IGFBP protease, which cleaves IGFBP-3, prevents its binding to IGF-1, and balances the free IGF-1 levels [17]. The balance of all these three, IGF-1, IGFBP-3, and IGFBP protease, determines free levels of IGF-1 in the blood (Figure 1) [18]. 

Since IGF-1 receptors are expressed in both normal and malignant colorectal epithelia, when activated by IGF-1, the receptor-ligand complex suppresses apoptosis and enables progression through the cell cycle by activating signal transduction pathways critical for cell growth and survival (Figure 1) [17,19,20]. Extensive epithelial proliferation and a wide zone of proliferation found in the colon of acromegalic patients are related to the levels of GH and IGF-1 [21]. As a result, IGF-1 has the ability to affect both pre-malignant and neoplastic phases. Furthermore, IGF-1 stimulates the synthesis of vascular endothelial growth factor, an angiogenic agent that promotes the development of blood vessels to support the colon cancer cell lines (Figure 1) [22]. Additionally, IGF-1 receptors are upregulated in the colon of acromegaly patients, which is vital to the survival of the mutated cells, giving legitimacy to the link between the IGF axis and neoplasia [23]. Above mentioned might be one of the probable pathogenic mechanisms by which IGF-1 contributes to the development and maintenance of colonic polyps in acromegaly patients. 

Gonzalez et al. conducted a case-control nested in a cohort study in 2017 further to support this putative function of IGF-1 in polyp development. The study revealed that high IGF-1 levels are significantly related to the formation of colonic polyps, indicating that acromegaly patients are at a high risk of acquiring colonic polyps [24]. Since colonic polyps can cause various abdominal symptoms, identifying individuals solely based on their clinical presentation can be challenging. Wei et al. conducted retrospective research at Beijing children’s hospital to determine the incidence of colon polyp symptoms in 487 patients. The study discovered that rectal bleeding was the most common symptom in most patients, with additional symptoms such as stomach discomfort, polyp protrusion from the anus, anemia, and diarrhea occurring in a few with the common site of polyps being the rectosigmoid colon, and the number of polyps being single or multiple [25]. However, in patients with acromegaly, colonic polyps could present somewhat differently. A study done in 2020 by Inayet et al. suggested that the most cited issue in patients with acromegaly is functional constipation. Such abdominal symptoms in patients with acromegaly may serve as a red flag for the physician, prompting him or her to suspect and begin screening [26].

Ochiai et al. published a case-control study in 2020 with 178 cases and 356 age and gender-matched controls to investigate the incidence of colon polyps in acromegaly patients. Following colonoscopy, 66.8% of acromegaly patients were discovered to have polyps, but only 24.2% of controls (healthy individuals) were found to have polyps (p=0.001). Acromegalic patients exhibited a higher number and larger size of polyps. The most prevalent location was the rectosigmoid area, indicating that individuals with acromegaly are at an increased risk of developing colorectal polyps than the general population (Table 1) [27]. In Turkey, Iliaz et al. conducted a case-control study in 2018 with 134 acromegaly patients as cases and 134 age- and gender-matched irritable bowel syndrome patients as controls. Following a colonoscopy and histopathological examination, it was discovered that the acromegaly patients had a substantially greater incidence of all types of colonic polyps (p=0.012). Histopathological examination revealed a higher incidence of hyperplastic polyps (p=0.004), indicating that the incidence of hyperplastic polyps was increased in Turkish individuals with active acromegaly (Table 1) [28]. Another study showed similar results as the studies mentioned above. It was conducted in Poland in 2010 with 31 acromegaly patients with a mean age of 46.3 +/- 11.9 years. Colon polyps were found in 13 individuals (41.9%) following colonoscopy. The results heavily suggested that colon polyp frequency was linked to the duration of uncontrolled acromegaly (p=0.01) (Table 1) [29]. It is also worth mentioning that, apart from polyps, colonic diverticula have also been reported in patients with acromegaly, which can be explained by high IGF-1 levels in these patients [30]. 

**Table 1:** Summary of included studies revealing a clinical correlation between acromegaly and colorectal polyps

Reference	Design	Population	Method	Diagnostic modality	Results	Conclusion
Ochiai et al. (2020) [27]	Retrospective matched case control	Patients with acromegaly diagnosed between april 2008 and september 2016 in toranomon hospital, Japan.	Cases-178 Controls-356 (Matched for age and gender)	Colonoscopy	66.8% of cases and 24.2% (p= 0.001) of controls were found to have polyps. The size and number were larger in the acromegaly (case) group.	Patients with acromegaly are at greater risk of developing colorectal polyps.
Iliaz et al. (2018) [28]	Case control study	Turkey	Cases-134 Controls-134	Colonoscopy followed by histopathological examination	The incidence of polyps in the acromegaly group was higher (p= 0.012) with an enhanced incidence of hyperplastic polyps (p= 0.004).	Turkish people with acromegaly had a higher incidence of hyperplastic polyps.
Krzentowska et al. (2010) [29]		Poland	Total patients-31. Mean age- 46.3 +/- 11.9 years	Colonoscopy	13 individuals (41.9%) were found to have polyps.	The length of uncontrolled acromegaly plays a role in the development of colon polyps (p= 0.01)

These studies give compelling evidence for a favorable clinical relationship between acromegaly and colorectal polyps. Colonoscopy and CT colonography are the two screening modalities. Once detected, polyps are very well treated with endoscopic management modalities such as polypectomy, endoscopic submucosal dissection (ESD), and endoscopic mucosal resection (EMR) if detected at an early stage. Endoscopic management modalities are used to treat polyps based on their size and histological features [31]. Polyps less than 5mm in size are too tiny to be surgically removed; instead, a yearly colonoscopy for three years is recommended [31,32]. They can potentially grow into life-threatening carcinoma if left untreated for an extended period. With the use of regular screening, polyps can be detected earlier and treated before developing into carcinomas. 

Acromegaly and colorectal carcinoma

The IGF-1 axis plays a vital role in carcinogenesis by promoting cellular turnover, leading to the accumulation of molecular alterations that influence colon carcinoma development [33]. Colon carcinoma can also be driven by RAS protein mutations that lead to increased IGF-1 signal transduction pathway activity, elevating gene expression and mitogenicity, promoting the adenoma-carcinoma sequence [34,35]. Tripkovic et al. conducted a study and demonstrated that individuals with colon carcinoma had elevated levels of circulating IGF-1 [36]. Zhang et al., on the other hand, conducted a similar study to investigate the role of IGF-1 and its receptor (IGF1R) in colorectal carcinoma and discovered comparable results that high circulating IGF1 levels and mucosal IGF1R expression may play a significant role in both the formation and development of colorectal carcinoma and may encourage the growth and malignant transformation of adenomatous polyps [37]. 

Battistone et al. recently published a case-control study that comprised 70 acromegaly patients and 128 healthy controls. Advanced neoplastic lesions were found in 22 (31.4%) of patients and nine (7.0%) of controls (p=0.0001, odds ratio (OR): 6.06); advanced adenomas were found in 18 (27.3%) and nine (7.0%) of patients and controls, respectively (p=0.0006, OR: 4.57), and colorectal carcinomas were discovered in four (5.7%) and zero (0.0%) of patients and controls, respectively (p=0.0063), indicating that acromegaly patients had a higher risk of developing colon cancer (Table 2) [38]. In 2018, Dal et al. published a cohort study and a meta-analysis of the literature to reinforce the cohort research’s findings. The study tracked a cohort of 529 acromegaly patients from the date of diagnosis with acromegaly until the first occurrence of a cancer diagnosis, death, emigration, or end of the study period, whichever came first, and discovered 81 cases of various malignancies (standardized incidence ratio (SIR) 1.1; 95% confidence interval (CI): 0.9 to 1.4). Colorectal cancer had a SIR of 1.4 (95% CI: 0.7 to 2.6). These results were backed up by a meta-analysis of 23 studies producing overall cancer SIR of 1.5 (95% CI: 1.2 to 1.8) with an increased SIR for colorectal cancer of 2.6 (95% CI: 1.7 to 4.0), showing modestly higher incidence rates in acromegaly patients, but it also highlighted the possibility of selection bias in certain previous studies (Table 2) [39]. Terzolo et al. assessed the SIRs of several kinds of cancer in acromegaly in 2017 using a multi-centered cohort analysis of 1512 acromegaly patients. After a 10-year follow-up, 124 individuals were diagnosed with cancer, with a substantially higher incidence of colon cancer (SIR: 1.67; 95% CI: 1.07-2.58, p=0.022), demonstrating a modest increase in cancer risk in acromegaly patients (Table 2) [40]. Wolinski et al. performed a case-control study in 2016; 200 patients and 145 controls were used in the study. Colon cancer was found in four (2.0%) of acromegaly patients and zero in the control group (p=0.14), suggesting a heightened risk of cancer in the acromegaly group (Table 2) [41]. 

**Table 2:** Summary of included studies revealing a clinical correlation between acromegaly and colorectal carcinoma OR: Odds Ratio SIR: Standardized Incidence Ratio CI: Confidence Interval

Reference (year)	Design	Population	Method	Results	Conclusion
Battistone et al. (2021) [38]	Case- control study	Patients with acromegaly from 15 Buenos Aires hospitals.	Cases- 70 Controls- 128 All of them underwent colonoscopy and histopathological examination.	1.Advanced neoplastic lesions seen in: Cases-22 (31.4%) Controls-nine (7.0%) (p=0.0001, OR: 6.06) 2.Advanced adenomas: Cases-18 (27.3%) Controls-nine (7.0%) (p=0.0006, OR: 4.57) 3.Advanced carcinomas: Cases-four (5.7%) Controls-zero (0.0%) (p=0.0063)	There is a high risk of colon carcinoma in acromegalic patients.
Dal et al. (2018) [39]	Cohort study	Population comprised the cumulative population of Denmark. Recruited data from the Danish National Patient Registry.	A cohort of 529 patients of acromegaly was followed from the date of diagnosis with acromegaly until the first occurrence of a cancer diagnosis, death, emigration, or end of the study period, whichever came first.	SIR for colorectal cancer: 1.4 (95% CI: 0.7 to 2.6)	The elevated incidence rate of colorectal cancer in acromegaly patients.
Terzolo et al. (2017) [40]	Cohort study	Italy	Assessed SIR of colorectal cancer in 1512 cases of acromegaly who were followed up for 10 years.	Incidence of colorectal cancer (SIR- 1.67; 95% CI: 1.07-2.58, p=0.022)	Risk of colorectal cancer in acromegaly patients is moderately elevated
Wolinski al. (2016) [41]	Case-control study		Cases- 200 Controls- 145	Colon cancer was found in four (2.0%) cases and zero controls (p=0.14).	There is a high risk of colon malignancy in acromegaly patients.

Kurimuto et al. conducted research in 2008 to investigate the prevalence of benign and malignant tumors in individuals with acromegaly. A retrospective chart analysis was done on 140 patients with active acromegaly who had visited an outpatient clinic (male/female 54/86, age 55 +/- 25-year, range 21-86). In 10 individuals, colon cancer was discovered. In comparison to the general population, the SIRs for colon cancer in acromegaly patients was 17.4 (95% CI: 4.74-44.55) for females and 19.0 (95% CI: 5.18-48.64) for males, indicating a substantial risk of colon cancer in acromegaly patients [42]. In 2008, Rokkas et al. published a meta-analysis assessing the probability of colorectal neoplasia in acromegaly patients. The study analyzed nine trials that included 701 patients with acromegaly and 1573 controls. The pooled findings revealed that individuals with acromegaly had an elevated risk of colon cancer (OR: 4.351; 95% CI: 1.533-12.354; Z=2.762, p=0.006), consistent with the higher risk of colorectal cancer reported by the research mentioned above [43]. Terzolo et al. conducted a cross-sectional study with 235 acromegaly patients in Italy in 2005. Acromegaly patients’ colonoscopic findings were compared to 233 individuals with nonspecific symptoms. Ten patients (4.3%) and two control participants (0.9%) had carcinoma (OR: 4.9; range-1.1-22.4), with a higher probability of colonic neoplasia occurring in younger acromegaly patients compared to age-matched controls, implying that acromegaly carries a moderate but definite elevated risk of colonic cancer that develops at a relatively young age than in the general population [44]. Matano et al. conducted a comparative investigation and found a similar outcome. In 2005, 19 acromegaly patients (cases) and 76 age, gender, and smoking status matched controls enrolled in the case-control study. The prevalence of cancer was more significant in acromegaly patients than in controls (p=0.05, OR: 9.8), demonstrating a link between acromegaly and colon carcinoma [45]. 

These findings provide convincing evidence for a favorable clinical connection between acromegaly and colorectal carcinoma. Having stated that, screening for colon pathologies in acromegaly patients becomes critical. The objectives of screening are to detect dysplasia before it advances to carcinoma, detect carcinoma before clinical symptoms appear, and prevent it from metastasizing. Direct visualization screening using colonoscopy and flexible sigmoidoscopy, and CT colonography are considered as more effective modalities for identifying colon neoplasia [46]. Alternative stool-based screening procedures that are less effective, such as fecal immunochemical testing and fecal occult blood tests, stool deoxyribonucleic acid (DNA) tests combined with fecal immunochemical tests, are generally avoided in these people [47]. Screening colonoscopy should not be postponed in individuals with acromegaly under the age of 45, which is the age at which screening is suggested in the average-risk group according to the U.S. Preventive Services Task Force (USPSTF) [48]. There is substantial agreement across endocrine scientific societies on needing a colonoscopy when acromegaly is diagnosed [49-52]. Repeat colonoscopy should be performed every five years if a colonic adenoma is discovered during screening and if acromegaly is not adequately biochemically managed. The timeframe for repeat colonoscopy changes according to the levels of GH and IGF-1. Follow-up colonoscopy should be conducted with stricter compliance in such patients than in the general population. On the other hand, surveillance colonoscopy is recommended every 10 years when acromegaly is biochemically controlled [47]. 

Limitations

Potential confounding factors for the development of colorectal neoplasia such as age, gender, insulin resistance, circulating insulin levels, diabetes mellitus status, hypertension status, body mass index, high fatty diet, family history, and geographical distribution have not been taken into account in all of the studies presented. 

## Conclusions

According to the studies covered in this article, despite patients with acromegaly having significant morphological alterations, the condition is also a causal factor for colorectal neoplasia such as colorectal polyps and colorectal carcinoma. IGF-1 is responsible for the majority of the pathological changes that take place in acromegaly. Keeping the aforementioned facts in mind, we attempted to emphasize colorectal neoplasia in patients with acromegaly in this review article to raise awareness among physicians to begin screening for colorectal neoplasia using direct visualization methods such as colonoscopy and CT colonography at the moment the patient is diagnosed with acromegaly. When such pathologies are identified at an early pre-malignant stage with the aid of screening methods, grave sequelae such as colonic carcinoma can be prevented with effective management, which has the potential to improve the quality of life and extend the life expectancy of acromegalic patients. We believe that this article can serve as a foundation for researchers to delve deeper into colorectal complications of acromegaly and explore the pathophysiology of this illness in depth. However, we believe that the link between acromegaly and colorectal neoplasia requires further large-scale research studies to be conducted while keeping confounding variables in mind in order to establish appropriate screening and management guidelines. 
